# AI in optimized cancer treatment: laying the groundwork for interdisciplinary progress

**DOI:** 10.1093/oxfimm/iqaf004

**Published:** 2025-05-12

**Authors:** Gabriel Kalweit, Evelyn Ullrich, Joschka Boedecker, Roland Mertelsmann, Maria Kalweit

**Affiliations:** Collaborative Research Institute Intelligent Oncology (CRIION), Freiburg, Baden-Württemberg, 79110, Germany; Department of Computer Science, University of Freiburg, Freiburg, Baden-Württemberg, 79110, Germany; Collaborative Research Institute Intelligent Oncology (CRIION), Freiburg, Baden-Württemberg, 79110, Germany; Department of Pediatrics, Experimental Immunology and Cell Therapy, Goethe University, Frankfurt am Main, Hessen, 60590, Germany; German Cancer Consortium (DKTK) and German Cancer Research Center (DKFZ), Partner Site Frankfurt, 60590, Germany; Frankfurt Cancer Institute (FCI), Goethe University, Frankfurt am Main, Hessen, 60596, Germany; Collaborative Research Institute Intelligent Oncology (CRIION), Freiburg, Baden-Württemberg, 79110, Germany; Department of Computer Science, University of Freiburg, Freiburg, Baden-Württemberg, 79110, Germany; IMBIT//BrainLinks-BrainTools, University of Freiburg, Freiburg, Baden-Württemberg, 79110, Germany; Collaborative Research Institute Intelligent Oncology (CRIION), Freiburg, Baden-Württemberg, 79110, Germany; Mertelsmann Foundation, Freiburg, Baden-Württemberg, 79104, Germany; Department of Hematology, Oncology and Stem Cell Transplantation, Medical Center University of Freiburg, Faculty of Medicine, University of Freiburg, Freiburg, Baden-Württemberg, 79106, Germany; Collaborative Research Institute Intelligent Oncology (CRIION), Freiburg, Baden-Württemberg, 79110, Germany; Department of Computer Science, University of Freiburg, Freiburg, Baden-Württemberg, 79110, Germany

**Keywords:** AI in oncology, interdisciplinary collaboration, optimized cancer treatment

## Abstract

The molecular complexity of cancer presents significant challenges to traditional therapeutic approaches, necessitating the development of innovative treatment strategies capable of addressing the disease’s dynamic nature and resistance mechanisms. Data-driven methodologies, particularly those employing Artificial Intelligence (AI), hold substantial promise by advancing a comprehensive understanding of the intricate molecular and cellular mechanisms underlying cancer and supporting the development of adaptive, patient-specific therapeutic strategies. Initiated through the Mertelsmann Foundation, the Collaborative Research Institute Intelligent Oncology (CRIION) in Freiburg im Breisgau, Germany, aims to drive progress in AI-driven oncology. CRIION fosters global collaboration through initiatives like the Intelligent Oncology Symposium and supports multidisciplinary projects designed to integrate AI innovations into clinical workflows.

## Introduction

The field of oncology is undergoing a paradigm shift, with advancements in AI playing a pivotal role in this transformation [1–3]. Cancer, a leading cause of global mortality, is projected to affect 35 million individuals annually by 2050 [4]. One of the foremost challenges in addressing this disease arises from its extraordinary molecular complexity. There are more potential combinations of cancerous mutations than atoms in the universe [5], highlighting the astronomical number of genetic variations that can drive cancer progression. Identifying the pathways responsible for specific mutations is further complicated by the foundational principles of cancer biology, as outlined by Douglas Hanahan and Robert A. Weinberg in their seminal work *Hallmarks of Cancer* [6, 7]. The complexity of this task is analogous to finding a needle in a haystack, posing substantial hurdles to developing targeted therapeutic strategies.

In practice, the search for the right pathway is often infeasible, and many cases, such as those of Acute Myeloid Leukemia (AML), follow a starkly defined course. AML, the deadliest form of leukemia, exemplifies the challenges of cancer treatment [8–10]. Despite aggressive therapeutic interventions, only about 30% of patients survive 5 years post-diagnosis, with survival dropping to 10% for those over 65 years of age. Initial treatment typically involves the rapid initiation of chemotherapy with the aim of achieving remission. For those who respond, a consolidation phase follows to prevent relapse, yet the prognosis remains grim. Approximately half of the patients relapse within the first year, and this figure rises up to 80% within 5 years [11]. These relapses represent critical junctures in the patient journey, underscoring the limitations of current therapeutic strategies and the immense complexity of addressing the underlying biology of the disease. Furthermore, resistance to chemotherapy significantly reduces treatment options and increases the likelihood of relapse, particularly in older patients where treatment efficacy is already limited.

This emphasizes the urgent need for adaptive, personalized treatments capable of evolving over time to address the dynamic nature of cancer. One promising avenue lies in leveraging AI to guide treatment strategies tailored to individual patients. While such an approach might initially appear overly ambitious and subject to significant regulatory hurdles, precedents already exist. For example, a medical device employing data-driven algorithms to adapt insulin delivery has received approval [12], demonstrating the feasibility of adaptive treatments in principle. Applying similar methodologies to oncology, despite the additional layers of complexity inherent in cancer therapies, offers a viable pathway forward. Initiated through the Mertelsmann Foundation, the Collaborative Research Institute Intelligent Oncology (CRIION) in Freiburg im Breisgau, Germany, has been established with the goal of advancing adaptive, AI-driven treatment strategies in oncology. By fostering interdisciplinary collaboration and integrating innovative AI solutions, CRIION aims to address the dynamic challenges of cancer care and contribute to a future where therapies are tailored to the individual needs of each patient.

## Roadmap for adaptive and personalized treatments

The development of adaptive treatment demands advancements at multiple levels. These include improvements in diagnosis, such as the detection and classification of cells and their states across multiple scales, encompassing molecular, cellular, and multicellular levels [13]. It also involves modeling transitions influenced by genetic variation, natural processes, or engineered perturbations. Prognosis focuses on the prediction of cell and (immuno-)therapy response dynamics, future states and complex tumor microenvironment interactions, agnostic to different drugs and dosages, guiding future data generation and in silico experimentation. Additionally, treatment strategies must integrate the ability to combine existing drugs effectively for individual treatment decisions, alongside the discovery and development of new drugs or neoantigen-based therapies. A critical component is the incorporation of temporal factors into treatment strategies, to allow therapies to evolve continuously as the disease progresses and adapt dynamically to emerging resistances. Across all these areas, efforts must span multiple data modalities, integrating established methods with innovations while also exploring entirely novel approaches. These efforts should aim to develop efficient and effective solutions that enable fast and robust readouts with feasible resource requirements, ensuring practical scalability and adaptability in real-world applications. This vision constitutes a broad roadmap with numerous incremental and transformative milestones to be achieved across these critical areas.

## The need for multidisciplinary collaboration

Achieving this ambitious goal requires the collaboration of diverse disciplines, including medicine and biology, hardware engineering, computer science and AI, while maintaining close supervision and engaging in formative discussions with fields such as ethics, law, and nursing sciences. However, each of these disciplines comes with its unique workflows and scientific cultures, necessitating the creation of a space where these perspectives can intersect, fostering mutual understanding and establishing a common language.

**Figure 1.**
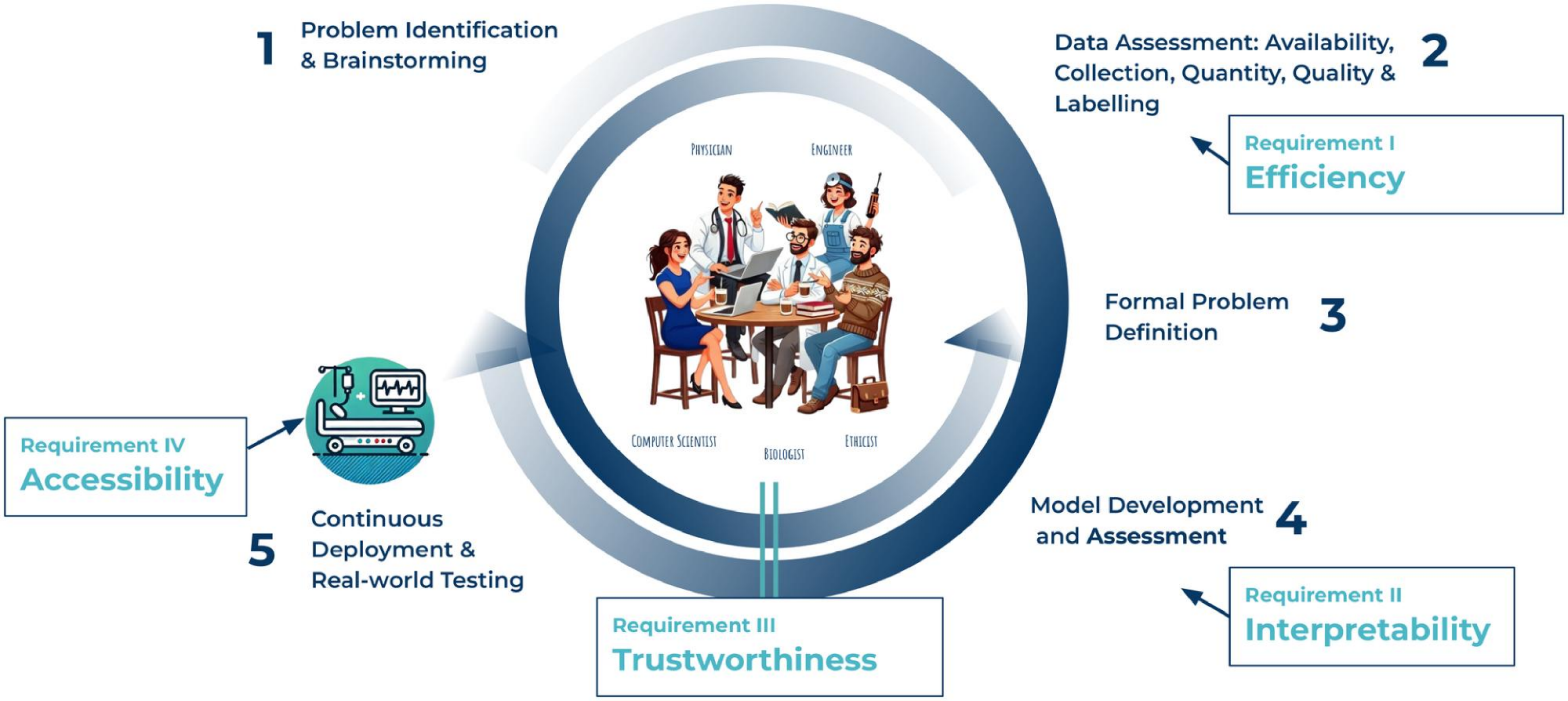
Interdisciplinary framework for advancing AI-assisted oncology. The framework outlines a five-step process involving physicians, biologists, computer scientists, engineers and ethicists to develop and implement Machine Learning solutions in Translational Oncology. Key steps include problem identification (Step 1), data assessment (Step 2, Requirement I), formal problem definition (Step 3), model development and assessment (Step 4, Requirement II), and continuous deployment emphasizing trustworthiness (Requirement III) and accessibility (Requirement IV, Step 5). This approach ensures clinically relevant, reliable, and widely adoptable solutions. The Figure contains AI-generated components created with DALL-E 2.

To foster mutual understanding and ensure close collaboration among all disciplines involved, an interdisciplinary framework (cf. Fig. 1) is essential to guarantee alignment among stakeholders [14]. This process begins with problem identification and brainstorming, where experts from various fields define key challenges and opportunities from their unique perspectives and expertise. The next step involves data assessment, focusing on evaluating data availability, quality, and quantity, as well as labeling practices and requirements. With these foundations in place, a formal problem definition is developed to align technical and clinical objectives.

Depending on the specific characteristics of the defined problem—such as the number of prediction or decision steps involved, the complexity of expert judgments to be captured, and the availability of labels and ground truth—expertise across the full spectrum of AI methodologies is brought together within CRIION. Depending on the required expressiveness and feasibility of the solution, approaches span from classical machine learning algorithms [15, 16] to sophisticated deep function approximators [17], and from unsupervised [18] and supervised [19] learning to (inverse) reinforcement learning [20, 21]. Strategic emphasis is placed on the transferability and robustness of solutions, e.g. by leveraging generalization capabilities of foundation models [22], the improvement and scalability of explainability methods [23, 24], and carefully balancing model expressiveness against computational cost. The integration of complementary modules—such as prediction, decision support, and uncertainty estimation—is pursued to build coherent system-level solutions capable of robustly tackling variability, uncertainty, and heterogeneity, while accounting for the safety-critical nature of clinical and oncological decision-making.

Following this, model development and assessment are conducted, measuring both objective performance criteria and human understanding of the results. Finally, the framework concludes with continuous deployment and real-world testing, where AI systems are integrated into clinical workflows and iteratively improved for robustness through feedback from real-world applications, held-out test sets, and fundamental unit tests within allowed fluctuations.

## Key desiderata for AI in oncology

Underlying this framework are four central desiderata:

1. *Trustworthiness*, emphasizing reliability, robustness, and safety in high-stakes medical applications. This includes respecting ethical values and principles [25, 26] as well as reproducibility through automation.
2. *Efficiency*, which aims to balance the data requirements of sophisticated AI systems with the challenges of data collection and labeling in the wet lab.
3. *Explainability*, to provide transparent and interpretable AI models [27] that foster trust among clinicians and patients.
4. *Accessibility*, ensuring that research and technologies can be implemented across diverse clinical settings and populations. Accessibility encompasses both AI applications and data management, enabling the sharing and use of datasets across different institutions. Supported initiatives, such as the FRAI.lab at the University Hospital Freiburg, focus on establishing robust medical data interfaces for hospitals, further enabling this goal.

By adhering to the above mentioned guiding principles, this interdisciplinary approach seeks to bridge the gap between cutting-edge innovation and meaningful patient impact in oncology.

## Targeted research projects addressing clinical challenges

Building upon the outlined roadmap and guided by the key desiderata, the Mertelsmann Foundation has funded several targeted research projects within CRIION. These projects, undertaken with research groups at the University Hospitals of Freiburg, Augsburg, Basel, Frankfurt, and Zurich, the University of Freiburg, the Universities of Applied Sciences in Furtwangen and Munich, as well as industrial partners such as Labmaite GmbH, are designed to address specific challenges in developing adaptive, personalized cancer treatments.

### Enhancing diagnosis and prognosis

At the molecular level, efforts focus on integrating diverse data modalities, such as detecting disease signatures in immune repertoires [15, 16, 28–30] and through liquid biopsies [31, 32]. These projects aim to develop diagnostic systems, exploring biomarkers that provide insights into immune responses and tumor genetics. At the cellular and multi-cellular levels, projects aim to enhance diagnostic precision by classifying stained cells across multiple scales [17, 22], using advanced imaging techniques and Machine Learning algorithms to achieve rapid readouts. These efforts contribute to the roadmap’s goal of improving diagnosis through detailed detection and classification of cells and their states. Expanding beyond stained fixed-cell images, CRIION is advancing into the realm of live-cell imaging [22], enabling the detection of dynamic cell states and transitions in live cells, which aligns with modeling transitions influenced by genetic variations and natural processes. Recognizing the importance of reducing the burden of data annotation for recorded images (posing a significant bottleneck in training AI models), there is a need to develop AI models that minimize data annotation requirements [18], making sophisticated AI systems more feasible in clinical settings where data is often scarce or expensive to obtain.

CRIION also aims towards forecasting cell dynamics over time. This predictive capability is crucial for prognosis, as it allows researchers to anticipate how cancer cells might evolve in response to treatments, thereby informing more effective therapeutic strategies. Since explaining complex AI prediction models can help identifying novel AI-based biomarkers, CRIION investigates the development of novel explainability methods for state-of-the-art AI models, such as (vision) transformers. By enhancing the transparency of AI decision-making processes, this supports the desideratum of building trustworthiness in AI systems among clinicians and patients. Further, hardware development of devices and platforms is required to support the real-time monitoring and analysis required for adaptive treatments, enhancing both the accessibility and practicality of these technologies in clinical environments [33].

### Treatment optimization

CRIION projects aim to create efficient active and offline learning methods for drug discovery [20], particularly in designing antibody sequences with desired properties. By incorporating asynchronous wet lab interactions, these approaches facilitate a more dynamic and responsive drug development process, aligning with the roadmap's vision of adaptive treatments that evolve over time. Understanding that technological advancements must be accompanied by ethical considerations, particularly for treatment, CRIION contributes to laying an ethical framework for AI in oncology. This effort ensures that the deployment of AI in cancer care adheres to ethical standards, addressing concerns such as patient privacy, data security, and informed consent, thereby reinforcing the desideratum of trustworthiness.

By anchoring these projects with junior professorships in core research areas and maintaining a strong connection with academia and industry partners, CRIION ensures that its efforts are both cutting-edge and grounded in practical applicability. Each project contributes a vital component to the overarching goal of developing adaptive, personalized treatments, bringing oncology closer to a future where care is more effective, precise, and responsive to individual patient needs. Translating research innovations into clinical practice is essential for improving patient outcomes in oncology. This process ensures that scientific discoveries and technological advancements directly benefit those affected by cancer.

## The Intelligent Oncology Symposium: a platform for global collaboration

CRIION aims at fostering international collaborations and has created a network to facilitate the exchange of ideas and the identification of important research directions. To strengthen this mission, the *Intelligent Oncology Symposium* was established as a bi-yearly event, providing a platform for interdisciplinary dialogue and global cooperation in AI-driven oncology. Over the course of two successful symposia, the events have welcomed several speakers from 9 different countries and more than 300 attendees, emphasizing its role as a hub for international knowledge sharing.

Building on the urgent need for adaptive, personalized cancer treatments, the symposium brought together expertise from diverse fields to address both foundational challenges and practical applications. Discussions explored how AI can optimize diagnostic precision and therapeutic decisions [34–38], offering novel solutions to improve patient outcomes in oncology. This naturally led to examining how computational tools can provide deeper insights into the molecular and cellular complexities of cancer [39–42], bridging gaps in our understanding of disease mechanisms. These insights set the stage for addressing key regulatory and ethical considerations [43–47], ensuring that AI solutions are both trustworthy and accessible for clinical use. The symposium also highlighted pathways for translating these advances into practice [48–52], emphasizing strategies to deploy AI effectively in real-world settings while accounting for the constraints of healthcare systems and patient needs.

By fostering this multidisciplinary dialogue, the symposium underscores CRIION’s vision of integrating cutting-edge AI innovations with clinical expertise to develop adaptive, scalable, and equitable approaches to cancer care.

## Conclusion: towards a future of adaptive cancer care

Artificial intelligence offers powerful tools to provide deeper insights into disease mechanisms of cancer. The roadmap presented here highlights the importance of multidisciplinary collaboration, combining expertise from medicine, biology, computer science, and engineering, while addressing ethical and regulatory considerations essential for clinical adoption. Initiated through the Mertelsmann Foundation, the interdisciplinary *Collaborative Research Institute Intelligent Oncology (CRIION)*, strives to create an open and creative research network at the intersection of Translational Oncology and Machine Learning for talents from around the globe in the heart of Freiburg, Germany. By being home to targeted projects and hosting initiatives like the Intelligent Oncology Symposium, CRIION facilitates the exchange of knowledge and the development of practical solutions. With a strong focus on accessibility, CRIION aims to ensure that AI technologies are both innovative and applicable across diverse healthcare settings, bringing us closer to improved outcomes and more equitable care for patients worldwide.

## Data Availability

No new data was generated or analysed for this manuscript.
